# Oxidative Stress in Pregnancy

**DOI:** 10.3390/biom13121768

**Published:** 2023-12-09

**Authors:** Konrad Grzeszczak, Natalia Łanocha-Arendarczyk, Witold Malinowski, Paweł Ziętek, Danuta Kosik-Bogacka

**Affiliations:** 1 Department of Biology and Medical Parasitology, Pomeranian Medical University in Szczecin, Powstanców Wielkopolskich 72, 70-111 Szczecin, Poland; konrad.grzeszczak@pum.edu.pl (K.G.); natalia.lanocha.arendarczyk@pum.edu.pl (N.Ł.-A.); 2 Department of Laboratory Diagnostics, Pomeranian Medical University, 70-111 Szczecin, Poland; 3 Faculty of Health Sciences, The Masovian. Public University in Płock, Plac Dąbrowskiego 2, 09-402 Płock, Poland; witold05@op.pl; 4 Department of Orthopaedics, Traumatology and Orthopaedic Oncology, Pomeranian Medical University, Unii Lubelskiej 1, 71-252 Szczecin, Poland; pawel.zietek@pum.edu.pl; 5 Independent Laboratory of Pharmaceutical Botany, Department of Biology and Medical Parasitology, Pomeranian Medical University in Szczecin, Powstanców Wielkopolskich 72, 70-111 Szczecin, Poland

**Keywords:** oxidative stress, pregnancy, trace elements

## Abstract

Recent years have seen an increased interest in the role of oxidative stress (OS) in pregnancy. Pregnancy inherently heightens susceptibility to OS, a condition fueled by a systemic inflammatory response that culminates in an elevated presence of reactive oxygen species (ROS) and reactive nitrogen species (RNS) in the circulatory system. The amplified OS in pregnancy can trigger a series of detrimental outcomes such as underdevelopment, abnormal placental function, and a host of pregnancy complications, including pre-eclampsia, embryonic resorption, recurrent pregnancy loss, fetal developmental anomalies, intrauterine growth restriction, and, in extreme instances, fetal death. The body’s response to mitigate the uncontrolled increase in RNS/ROS levels requires trace elements that take part in non-enzymatic and enzymatic defense processes, namely, copper (Cu), zinc (Zn), manganese (Mn), and selenium (Se). Determination of ROS concentrations poses a challenge due to their short half-lives, prompting the use of marker proteins, including malondialdehyde (MDA), superoxide dismutase (SOD), glutathione peroxidase (GPx), glutathione reductase (GR), catalase (CAT), and glutathione (GSH). These markers, indicative of oxidative stress intensity, can offer indirect assessments of pregnancy complications. Given the limitations of conducting experimental studies on pregnant women, animal models serve as valuable substitutes for in-depth research. This review of such models delves into the mechanism of OS in pregnancy and underscores the pivotal role of OS markers in their evaluation.

## 1. Introduction

Over the last five years, the scientific community has observed a significant surge in investigations probing the role of oxidative stress (OS) in the initiation and progression of many diseases (PubMed keyword: Oxidative stress, yielding 118,239 results as of 6 October 2023). A substantial part of these studies is devoted to unraveling the implications of reactive oxygen species (ROS) on the trajectory of pregnancy and its impacts on fetuses and neonates (PubMed keyword: Oxidative stress ANDI Pregnancy, yielding 3117 results as of 6 October 2023). Nonetheless, the intricate mechanisms responsible for the onset of pathophysiological alterations in response to ROS remain largely elusive.

Therefore, the aim of this paper is to elucidate the importance of ROS and reactive nitrogen species (RNS), as well as their modulatory effects on pregnancy. Additionally, we scrutinize the repercussions of OS on the maternal and fetal physiological state during pregnancy, with an emphasis on identifying potential biomarkers that could be instrumental in mitigating the risk of complications associated with pregnancy and childbirth. Our analysis is based on the analysis of scientific articles sourced from reputable databases such as PubMed, Embase, and the Web of Science. This review encompasses comprehensive reviews and original research articles written in English and published in peer-reviewed journals. We excluded brief communications, case reports, and the gray literature (e.g., conference proceedings and abstracts). No restrictions were imposed on the publication date. Upon application of these criteria, a total of 172 papers were shortlisted for review.

## 2. Reactive Oxygen and Nitrogen Species

RNS and ROS are generated during various biological processes. When released in physiological quantities, they function as mediators and regulators, ensuring proper cellular functioning [1]. RNS include nitric oxide (NO·) and peroxynitrite (ONOO^−^) [2]. ROS comprise superoxide radical anion (∙O_2_^−^), hydrogen peroxide (H_2_O_2_), hydroxyl radical (∙OH), hydroperoxyl radical (HOO∙), singlet oxygen ((1)O_2_), and peroxyl radical (ROO∙) [3]. 

The primary ROS generated during oxygen metabolism is the superoxide anion, which is highly reactive and cytotoxic. Under the influence of superoxide dismutase (SOD), it catalyzes the disproportionation to dioxygen and a significantly less reactive product—H_2_O_2_, thereby safeguarding cells from toxic oxygen respiration products [4]. The one-electron reduction in O_2_ to ∙O_2_^−^ and its dismutation to H_2_O_2_ occur during mitochondrial respiration. H_2_O_2_ is produced in mitochondria (superoxide dismutase reaction) and peroxisomes (acyl-CoA oxidase reaction). Mitochondria are also involved in generating NO via nitric oxide synthase (NOS). ∙O_2_^−^ and NO react to form peroxynitrite (ONOO^−^), a potential source of ∙OH [5], which forms in the presence of metals, including copper (Cu) and iron (Fe), and H_2_O_2_ (Fenton reaction). The non-enzymatic Fenton reaction is the degradation of H_2_O_2_ catalyzed by Fe^2+^, resulting in ∙OH and occurring in the endoplasmic reticulum [6].

Other non-mitochondrial reactions include the respiratory burst of phagocytic cells, which are sources of ∙O_2_^−^. In inflammatory states, according to the stress-induced premature senescence (SIPS) theory, sublethal doses of various stressogenic agents, including H_2_O_2_, exhaust the replicative potential of proliferating cells and induce the accumulation of aging cells, which may be responsible for creating a micro-inflammatory state and activating phagocytic cells. Another example of a non-mitochondrial reaction is the reaction occurring in peroxisomes with β-oxidation of fatty acids, generating H_2_O_2_ [7].

ROS exhibit higher reactivity than molecular oxygen in the ground (triplet) state. They can be generated endogenously or exogenously from numerous sources. The endogenous sources include the mitochondrial respiratory chain, the electron transport chain, the microsomal electron transport chain, oxidant enzymes (xanthine oxidase, cyclo-oxygenase), phagocytes, and cellular auto-oxidation of Fe^2+^ and epinephrine [8,9]. Exogenous sources include alcohol, tobacco smoke, poor diet, intense physical exertion, low temperatures, stress, injuries, heavy metals, transition metals, industrial solvents, pesticides, benzopyrene, radiation, certain drugs like halothane and paracetamol, and bacterial and viral infections [9].

Under physiological conditions, the production of ROS is tightly regulated by the body through the actions of enzymatic and non-enzymatic defensive mechanisms. However, the impact of ROS on cells largely depends on their concentration and duration of action. A brief increase in ROS production is usually well-tolerated by cells and typically results in an enhanced defensive response. However, an intense or prolonged state of OS, triggered by pathogenic factors or harmful external factors, induces damage to cellular components [10].

ROS participate in numerous processes, including muscle contraction, hormone secretion, immune system function, and vascular tension regulation. ROS influence cell growth and differentiation, growth factor activation, mitogenic response, extracellular matrix production modulation, and cell apoptosis. Moreover, reactive oxygen forms cause NO inactivation, pro-inflammatory gene stimulation, and activation of numerous kinases [11].

ROS play a critical regulatory role through various signaling transduction pathways in folliculogenesis, corpus luteum oocyte maturation, and feto-placental development [12,13]. During pregnancy, ROS are naturally produced during implantation, proliferation, differentiation, and trophoblastic invasion processes [14]. Their increased production is associated with placental function, among other things [15]. In the first trimester of pregnancy, the oxygen concentration in the placenta is low as it is not yet connected to the mother’s circulation, which leads to the generation of ROS that stimulate cell proliferation and angiogenesis, including the production of hypoxia-inducible factors (HIF), vascular endothelial growth factor (VEGF), and placental growth factor (PGF) [15,16,17]. In addition, nitric oxide (NO) contributes to maintaining vascular tension to increase blood flow in the uterus [18].

## 3. Oxidative Stress

Oxidative stress (OS) is caused by an imbalance between the production and accumulation of free radicals and the capacity of a biological system to detoxify these reactive products [17,19]. It is caused by increased levels of ROS and/or RNS or a decrease in antioxidant defense mechanisms, which can lead to chronic inflammation [20,21,22]. Generated ROS, including ∙O_2_^−^, H_2_O_2_, and ∙OH, cause damage to proteins and DNA and induce lipid peroxidation (LPO), which can result in the disturbance of membrane integrity and changes in DNA structure, leading to mutations or cytotoxic effects affecting cellular metabolism [23]. OS can be a direct or indirect cause of several disease conditions, such as diabetes mellitus, neurodegenerative disorders (Parkinson’s disease, Alzheimer’s disease, and multiple sclerosis), cardiovascular diseases (atherosclerosis and hypertension), respiratory diseases (asthma), cataract development, rheumatoid arthritis, and various cancers (colorectal, prostate, breast, lung, and bladder cancers) [9,24,25].

Free radicals affect various reproductive processes. For example, gametes are extremely sensitive to damage from ROS and need to be protected to maintain the survival of the species. OS can affect sperm structure and function, including decreased sperm viability, motility, number, and fertilization potential, which can lead to infertility [26,27].

OS is also considered to be responsible for the initiation or development of pathological processes affecting human female reproductive processes [27,28]. In the follicular fluid, ROS play an important role in the modulation of oocyte maturation, folliculogenesis, ovarian steroidogenesis, luteolysis, and ovulation in human females [29]. OS can lead to the occurrence of endometriosis, polycystic ovary syndrome, premature ovarian failure, and unexplained infertility in human females [15,30,31]. Furthermore, it has been linked to the adverse effect of repeated ovarian stimulation on the reproductive capabilities of mice [32], as well as to the developmental potential of oocytes under in vitro conditions [33] or in response to aging in human and animal models [34,35]. 

Pregnancy is a period of physiological and physical disturbance (adaptation to maintaining the growing fetus and preparation for childbirth and breastfeeding) in order to maintain the proper homeostasis of the mother’s body [36]. It is characterized by many physiological changes, resulting in increased basal oxygen consumption and changes in energy substrate usage by various organs, including the feto-placental unit [37]. Pregnancy is also associated with increased susceptibility to OS generated by the systemic inflammatory response [38,39], which plays a significant role during pregnancy, normal childbirth, and the initiation of preterm birth [40,41,42,43]. The systemic inflammatory response in pregnancy leads to the activation of peripheral granulocytes, monocytes, and lymphocytes during the third trimester, which produce large amounts of ROS [20,44]. 

Common disorders during pregnancy, such as endothelial cell dysfunction, are likely caused by ROS, which attack cell membrane phospholipids and react with polyunsaturated fatty acids, creating LPO and causing cell damage [45]. 

A state of excessive oxidation can lead to oxidative damage at the cellular/biochemical level involving a variety of biomolecules, including nucleic acids, proteins, lipids, and carbohydrates, even in pregnant women, but the mechanisms are very complex and require further research [46]. 

Spontaneous DNA mutations that accelerate genome instability have been found to be more common under conditions of oxidative stress [47]. OS is associated with changes in the DNA methylation pattern, with subsequent effects on fetal programming [48]. In addition, DNA methylation regulates gene expression without altering the DNA sequence and is induced by environmental stimuli [49]. Some studies of global methylation levels in the placenta and cord blood of women with and without gestational diabetes, pre-eclampsia, and obesity have suggested that maternal metabolic problems during pregnancy may influence the epigenome in the offspring [49,50]. Studies in animal models have shown that the intensity of diabetes is related to the level of oxidative DNA damage. It is possible that hyperglycemia has effects at the DNA level that extend beyond the pregnant mother [51].

The main source of ROS during pregnancy is the placenta [20], which, from early pregnancy, affects the mother’s homeostasis. Initially, the placenta has a hypoxic environment [52]. The mitochondria-rich placental activity and high maternal metabolism result in the production of a high level of ROS, mainly ∙O_2_^−^ and NO, which are important for placental blood perfusion and fetal nutrition [38,53]. 

**Figure 1:**
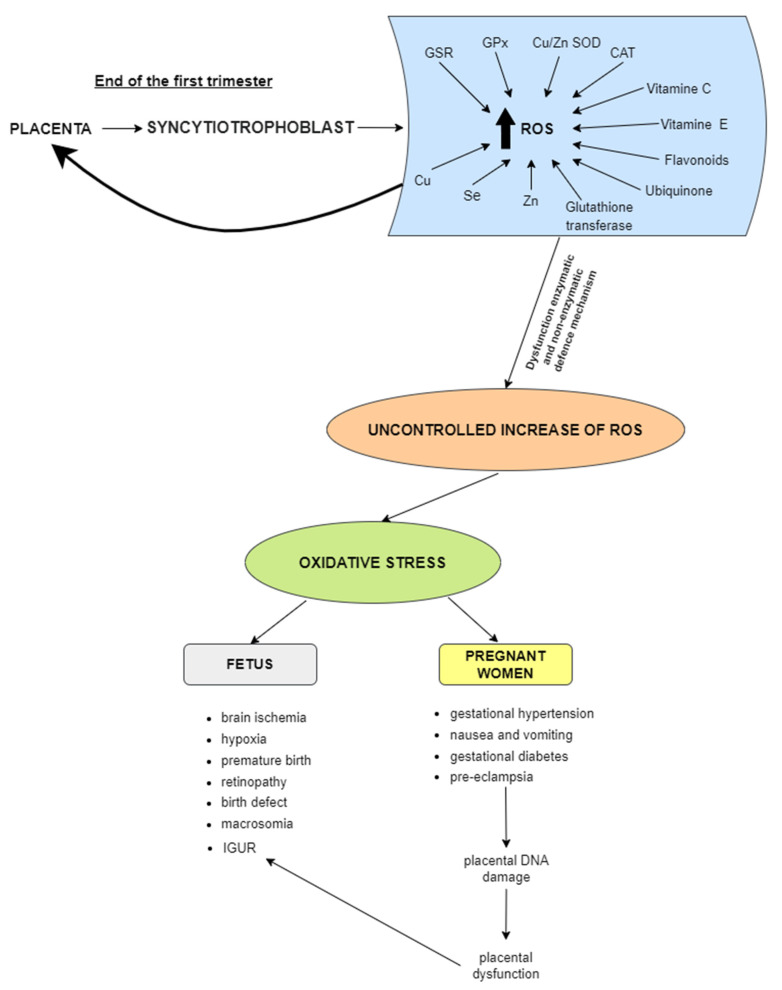
At the end of the first trimester, when the placenta is fully formed, there is a threefold increase in oxygen concentration, which leads to increased levels of ROS, mainly in the syncytiotrophoblast. Under physiological conditions, these processes are tightly controlled by the body as a result of enzymatic (GSR, glutathione reductase; GPx, glutathioneThe contents of this figure are not legible. Please replace the image with one of a sufficiently high resolution (min. 1000 The contents of this figure are not legible. Please replace the image with one of a sufficiently high resolution (min. 1000 pixels width/height, or a resolution of 300 dpi or higher).pixels width/height, or a resolution of 300 dpi or higher). peroxidase; SOD, superoxide dismutases; CAT, catalase) and non-enzymatic (glutathione, ubiquinone, vitamins E and C, flavonoids, zinc (Zn), selenium (Se), and copper (Cu)) defense mechanisms. Uncontrolled elevated levels of reactive oxygen species (ROS) and/or reactive nitrogen species (RNS) or a decrease in antioxidant defenses can lead to oxidative stress. Oxidative stress can be a direct or indirect cause of several conditions in fetuses (brain ischemia, hypoxia, premature birth, retinopathy, birth defects, macrosomia, and intrauterine growth restriction (IUGR)) or pregnant women (gestational hypertension, nausea, and vomiting, gestational diabetes, and pre-eclampsia, which lead to placental DNA damage and dysfunction resulting in IUGR).

By the end of the first trimester, the placenta is fully developed, and there is a threefold increase in oxygen concentration, leading to an increase in ROS levels, primarily in the syncytiotrophoblast (Figure 1). This process is fully regulated via the production of hypoxia-inducible factor 1 (HIF-1α) and the expression of genes encoding antioxidant enzymes, including heme oxygenase 1 and 2 (HO-1 and HO-2), copper–zinc superoxide dismutase (Cu/Zn-SOD), CAT, and glutathione peroxidase (GPx) [15,16,17]. Under physiological conditions, this is under strict control of the body due to the action of enzymatic and non-enzymatic defense mechanisms [54]. Based on the results of studies on pregnant women and using laboratory animals, it was noted that when OS exceeds the antioxidant defense of the placenta, oxidative damage can spread to distal tissues and can lead to many complications and abnormalities during pregnancy [15,16,17]. Accumulation of ROS leads to underdevelopment and abnormal placental function, which in turn causes disorders in the supply of oxygen and nutrients to the fetus [55,56]. This can cause the adhesion of leukocytes and platelets to the endothelium, as well as the release of cytokines and antiangiogenic factors. In inflammation, generalized vasoconstriction and increased resistance in the placental circulation may be due to a reduction in uteroplacental blood flow and placental dysfunction [57].

OS disrupts placental function and can alter fetal growth through various pathways, including modulation of key nutrient transporters such as Slc2a1 or Slc38a1 and cell death [58,59,60]. It can cause complications during pregnancy, such as embryonic resorption, recurrent pregnancy loss, intrauterine growth restriction, and fetal death [61,62].

OS and inflammatory responses are more pronounced in pre-eclampsia [45], which can lead to low birth weight and fetal developmental abnormalities [63]. Mothers in vaginal delivery and their newborns experience a higher OS than those who undergo elective cesarean sections for delivery [64]. 

It should be noted that during human preimplantation embryo development, ROS and RNS play a key role in regulating redox responses for optimal embryonic development [65]. ROS/RNS are required to regulate gene expression and signal transduction pathways important for normal embryonic development [66]. Unfortunately, the in vitro culture of embryos increases ROS production due to external factors, including the composition of the culture medium and laboratory culture conditions (temperature, humidity, and composition of the culture medium) [67]. This can lead to the uncontrolled growth of ROS, be detrimental during implantation and the development of assisted reproduction [68], and affect epigenetic and genetic changes in the embryo. This can result from the arrest of embryonic development, DNA damage, and the induction of apoptosis [69]. In addition, RNS are involved in the formation of the ONOO- [70] molecule, which can cause DNA strand breaks [71], leading to chromosomal abnormalities and developmental defects [72].

It is worth mentioning that low oxygen tension plays a key role in reducing high levels of harmful reactive oxygen species (ROS) in cells, influencing embryo gene expression, helping embryo glucose metabolism, and accelerating embryo development to the blastocyst stage [73]. It has been suggested that culture under reduced oxygen tension is critical for maintaining physiological embryo development and increasing reproductive competence. The current in vitro fertilization (IVF) laboratory program uses a 20% oxygen concentration supplemented with 5% CO_2_ [74]. In vivo studies have shown that oxygen tension measured in the mammalian oviduct varies between 2% and 8% [75], and, following this information, laboratory in vitro fertilization (IVF) studies have been conducted in a 5% O_2_ environment [76]. Due to the small differences in observed outcomes between embryos incubated at different oxygen concentrations, the researchers concluded that it may be premature to change the oxygen concentration in this procedure, and further studies should be conducted. Sciorio and Smith [77] came to a similar conclusion in their review, adding that further reductions in oxygen may be necessary to achieve excellent blastocyst formation and increase the policy of elective single embryo transfer. Van Montfoort et al. [78] studied the effects of 5% and 20% oxygen concentrations on human IVF embryo culture. They found that culturing embryos for 2 days in a 5% oxygen atmosphere had a beneficial effect on the percentage of live births, while there were no differences in the birth weight of live-born singletons between groups. Kelley and Gardner [79], studying mouse embryos in vitro, showed a detrimental effect of a 20% oxygen concentration on fetal and placental growth compared to embryos cultured under 5% oxygen. This is confirmed by Chen et al. [80], who conducted a study on a large group of patients undergoing IVF (*n* = 31,566). They observed that the birth weight of day 3 embryos cultured in 20% oxygen was significantly higher than the 5% oxygen group. Gelo et al. [81], in a prospective randomized study of a group of 393 patients, showed that culturing embryos in low oxygen (5%) produced more blastocysts and was, therefore, a better alternative for embryo selection, resulting in higher pregnancy rates. On the other hand, a study by Rendón Abad et al. [82] in a group of 1125 oocyte donations showed no differences in birth weight, birth length, head circumference, or 1 min Apgar score between culturing embryos under 6% and atmospheric oxygen concentration. 

It is very important when culturing embryos to mimic in utero conditions accurately. A description of normal mouse embryos ex utero from pre-gastrulation to advanced organogenesis was presented in Aguilera-Castrejon et al. [83]. They were cultured in conditions ranging from 5% to 21% O_2_ using three-dimensional rotating bottles and another using a combination of static and rotating bottle culture platforms [83]. These methods pave the way for the creation of synthetic embryos, provide new opportunities for science, and could lead to a reduction in the number of animals used in experiments.

During pregnancy, OS is closely associated with nausea and vomiting, and through changes in lipid metabolism, it indirectly affects gestational diabetes and fetal macrosomia. It also intensifies the tissue damage associated with diabetes. Through pre-eclampsia and pregnancy hypertension, OS increases the risk of premature delivery and maternal mortality [84]. Pre-eclampsia and the associated OS can damage placental DNA, which is probably associated with the disruption of its function and inhibition of fetal growth.

The perinatal period is important for maintaining a balance between the production of free radicals and the functional incompetence of the fetal and neonatal antioxidant systems. The values of OS indicators just after birth are elevated in both the mother and the child, and in the following few days in the newborn, they continue to rise. It has been shown that mother’s milk contains a proportional amount of antioxidants to the child’s deficiency, which may indicate its protective role in reducing OS [85].

The impact of the mode of delivery on the level of OS is still being researched. Fogel et al. [86] compared the level of OS in newborn humans who were born vaginally and through cesarean section. The study, examining the susceptibility of umbilical blood lipids to Cu-induced peroxidation, showed an elevated level of OS regardless of the mode of delivery. Vakilian et al. [87] compare both modes of delivery in humans using thiobarbituric reactive substances (TBARS) as markers of LPO, total antioxidant power (TAP), and total thiol molecules (TTM) in the blood of mothers and their newborns, showing that natural childbirth causes an increase in OS compared to cesarean section. Sgorbini et al. [88] found the same relationship in animal models when studying reactive oxygen metabolites (d-ROMs) and biological antioxidant potential (BAP), noting the effective antioxidant defense of newborns, who often coped with ROS better than their mothers.

However, the opposite relationship in humans was shown by Mutlu et al. [89], analyzing OS using total antioxidant capacity (TAC), total oxidant status (TOS), OS index (OSI), and lipid hydroperoxide (LOOH) levels, and by Şimşek et al. [90], who studied total antioxidative status (TAS), total oxidative status (TOS), oxidative stress index (OSI), malonyldialdehyde (MDA), and glutathione peroxidase (GSH) levels in humans. They noticed that natural childbirth was associated with decreased OS compared to a cesarean section.

Saphier et al. [91], based on the studies of other authors, also did not find significant differences in the level of OS between uncomplicated natural birth and planned cesarean section in human females. Moreover, Hung et al. [92] found that childbirth is associated with increased placental OS and affects maternal OS, and natural childbirth shows different OS indicators compared to cesarean section in human females.

Abnormal weight and weight gain in women are risk factors for oxidative stress [93]. This may be related to low levels of adipocytes, which are responsible for secreting adiponectins with antioxidant properties. A meta-analysis by Solis-Paredes et al. [94] found that women with gestational diabetes mellitus (GDM) have lower adiponectin levels than women without GDM. The study also showed that adiponectin levels are lower in women with GDM who have abnormal weight gain compared to those with adequate or inadequate weight gain. Subsequently, Solis-Paredes et al. [95] confirmed that women with abnormal weight gain have lower adiponectin levels than women with normal weight. In addition, they showed that reduced adiponectin levels may indicate weaker protection against ROS.

An elevated level of OS has been found in patients with gestational diabetes mellitus (GDM) [96,97]. Increased OS load may be responsible for the increased risk of pre-eclampsia and fetal developmental defects [98]. Pre-eclampsia and pregnancy hypertension are major causes of maternal mortality and morbidity and are often the causes of premature childbirth.

OS is further amplified by smoking [99], which has been proven in many studies showing that tobacco smoke carries over 1000 free radicals and enhances both basic and induced LPO [100,101,102]. This also applies to e-cigarettes, which adversely affect the endothelial network by inhibiting the promotion of OS and the adhesion of immune cells [103].

It has been found that prenatal OS may be accompanied by the low birth weight of the newborn [104]. Newborns undergo a number of physiological changes that significantly increase both the production of ROS and the possibility of OS occurrence [105,106]. Healthy infants are able to adapt to these changes, but premature and sick newborns are more susceptible to the negative impact of OS due to their immature endogenous and insufficient exogenous antioxidant protection [107,108]. An increasing effect of OS in preterm infants was observed if perinatal conditions (e.g., pre-eclampsia, hypoxia, and respiratory failure) or treatment (e.g., oxygen therapy) were present, which reduced their antioxidant capacity and additionally increased ROS production [109,110]. ROS play a role in the pathogenesis of many newborn diseases, such as retinopathy of prematurity, brain hypoxia and ischemia, intraventricular hemorrhage, and chronic lung disease [109,110].

## 4. Antioxidant System

Free radicals are neutralized via the antioxidant defense system. They are present in small concentrations and significantly prevent the oxidation of substrates [111,112,113]. Enzymatic and non-enzymatic antioxidants have been distinguished based on their activity in intracellular and extracellular compartments. Enzymatic antioxidants include SOD, GPx, catalase (CAT), glutathione transferase, and glutathione reductase (GSR) [38].

In mammals, SOD consists of three isoforms: the cytoplasmic Cu/ZnSOD (SOD1), the mitochondrial MnSOD (SOD2), and the extracellular Cu/ZnSOD (SOD3) [114]. Gpx reduces H_2_O_2_ and LPO to water and fatty alcohols, then GSH to glutathione disulfide (GSSG). CAT catalyzes the conversion of H_2_O_2_ to water and molecular oxygen, thus protecting cells from the harmful effects of H_2_O_2_ produced in the cell. This enzyme is most effective during increased OS when GSH or GPx levels are reduced. Reduced glutathione plays a major role in regulating the intracellular redox state of cells, as it is the main source of reduction equivalents. Thioredoxin reductase is responsible for thiol-dependent reduction processes in the cell. Glutathione S-transferase and H_2_O_2_ can form spontaneously or through SOD-catalyzed dismutation of •O_2_^−^: 2•O_2_^−^ + 2H^+^ → H_2_O_2_ + O_2_. Sulfur carriers are sensors in redox signaling pathways that control and integrate metabolic pathways. The three main redox controls responsible for regulating these carriers are thioredoxins, GSH/GSSG, and the redox couple cysteine/cystine (Cys/Cyss) [115].

Non-enzymatic antioxidants, including vitamin C, vitamin E, glutathione, ubiquinone, flavonoids, and antioxidant cofactors such as selenium (Se), zinc (Zn), and copper (Cu), are capable of removing, capturing, or inhibiting the formation of ROS [113]. These substances inhibit the degree of oxidation of molecules and cause these radicals to transform into inactive derivatives. The non-enzymatic line of antioxidant defense includes low-molecular-weight molecules, such as glutathione, uric acid, vitamin A (retinoids), carotenoids, and beta-carotene, which have high antioxidant activity as they trap free radicals. In addition, α-tocopherol (vitamin E), a fat-soluble free radical chain-breaking antioxidant due to the presence of a hydroxyl group (-OH) in its structure, is an effective hydrogen donor. Ascorbic acid (vitamin C) acts as a hydrogen donor, reverses the oxidation process, and can act both as an antioxidant and as a pro-oxidant [116]. Bilirubin, lipoic acid, albumin, ferritin, ceruloplasmin, and transferrin also exhibit antioxidant properties and may indirectly reduce or inhibit the generation of reactive forms [11].

During pregnancy, disturbances in the oxidative-antioxidant balance and a deficiency of antioxidants can affect fetal development. The human maternal status of antioxidant vitamins during pregnancy can impact fetal development [117]. It has been found that the antioxidant defense system is at a lower level in human patients with spontaneous miscarriages compared to women who have not experienced miscarriages [118]. Hernández-Trejo et al. [119] showed that maternal obesity influences OS during human pregnancy. SOD activity was found to be significantly higher in overweight/obese mothers compared to normal-weight mothers. No such relationship was seen in the newborns of overweight and normal-weight mothers, as the newborns had similar SOD activity. It was observed that maternal obesity influences OS and metabolism during pregnancy, thereby affecting the placenta and fetal growth, and that it may also impact the activation of the immune system.

## 5. Trace Elements and Oxidative Stress

Trace elements form a non-enzymatic defense line against OS. They are components of antioxidant enzymes and participate in the enzymatic mechanism [25]. Their deficiency is associated with increases in markers of oxidative damage, including DNA oxidation, protein oxidation, and LPO.

### 5.1. Copper

Cu plays a vital role in maintaining overall health, including reproductive health [120,121]. It acts as an essential cofactor for numerous enzymes involved in metabolic reactions, angiogenesis, and oxygen transport. Cu also influences the proper functioning of metallothionein and glutathione while affecting the activity of specific enzymes such as Cu/ZnSOD, ceruloplasmin, catalase, and peroxidases. Inadequate Cu levels in the body can lead to decreased enzyme activity, while excessive Cu concentrations, characteristic of transition elements, can promote OS [122].

Recent studies have highlighted the impact of serum Cu concentrations on complications in early pregnancy. Women with higher serum Cu concentrations are more prone to experience complications during the first trimester compared to those with lower concentrations. Inadequate Cu nutritional status impairs antioxidant mechanisms, whereas excessive concentrations stimulate the production of reactive oxygen species/reactive nitrogen species [123]. 

The precise relationship between Cu and OS levels is not yet fully understood. A recent study by Rak et al. [124] revealed a negative correlation between OS levels in male newborns and the concentration of Cu in maternal serum, which suggests a potential influence of Cu on OS during pregnancy, emphasizing the need for further research in this area.

### 5.2. Iron

Fe, an essential element found in substantial amounts in the placenta, possesses the potential to induce OS by generating ROS, which can cause damage to cells and tissues [37,122]. Acting as a catalyst in Fenton or Fenton-like reactions, Fe facilitates the conversion of ROS into highly reactive hydroxyl radicals (•OH) by reacting with H_2_O_2_ [125,126,127]. Consequently, Fe has the capacity to inflict various forms of oxidative damage to DNA, proteins, and cells.

In the context of pregnancy, Fe supplementation has been observed to heighten OS, as evidenced by increased levels of MDA in the serum of mothers and the placenta [128]. Similarly to Cu, Fe serves as a crucial cofactor in essential processes like oxygenation, reduction reactions, and antioxidant metabolism [129]. Both Fe deficiency and excess during pregnancy have been linked to adverse outcomes for fetal development [130]. Notably, prenatal Fe supersaturation has been associated with an elevated risk of miscarriage, prematurity, low birth weight, and being small for gestational age (SGA), with OS identified as one of the potential underlying mechanisms [130].

In pregnant women without Fe deficiency anemia (IDA), prophylactic Fe supplementation has been shown to induce OS and compromise the overall antioxidant capacity of the body [124,131]. Specifically, Rak et al. [124] established a correlation between excessive Fe concentrations exceeding 400 μg/dL in the blood of pregnant women and elevated levels of OS in neonates.

### 5.3. Zinc

Zn, an essential trace element, plays a pivotal role in various aspects of pregnancy, including embryogenesis, fetal growth, and development [132]. Inadequate Zn levels have been linked to adverse outcomes such as reduced implantation rates, abnormal ovarian development, impaired ovarian follicular growth, compromised oocyte maturation, and an increased risk of spontaneous abortions [133,134]. Maternal Zn deficiency during pregnancy also poses risks of low birth weight and infants being small for gestational age [135]. Furthermore, Zn deficiency has emerged as a potential risk factor for the development of pre-eclampsia [136].

Zn acts as an effective antiradical and anti-inflammatory agent. It forms chelates with sulfhydryl groups of proteins, providing protection against pro-oxidative processes. It shields cell membranes from peroxidation by displacing Cu and Fe ions from their membrane binding sites [25]. Additionally, Zn plays a vital role in the synthesis of antioxidant enzymes and serves as a catalyst for several enzymes involved in lipid, carbohydrate, and protein metabolism. Notably, Zn, in conjunction with Cu, serves as a cofactor for Cu/Zn-SOD, whose activity is compromised under Zn-deficient conditions [137].

Moreover, Zn exerts influence on the activity of other antioxidant enzymes. It displays catalytic functions for alkaline phosphatase and carboxypeptidase. By virtue of its antioxidative properties, Zn effectively hampers the generation of highly reactive •OH and superoxide anions (O2•^−^). Furthermore, Zn actively participates in the synthesis, storage, and release of insulin, underscoring its crucial role in the pathogenesis of type 2 diabetes, atherosclerosis, and metabolic syndrome [138,139,140].

In the bloodstream, Zn primarily binds to albumin (60%) and transferrin (10%), with the remaining fraction existing in its free form. Maintaining adequate Zn levels is critical for preserving normal reproductive health, as diminished amounts have been associated with serious maternal-fetal consequences, including postpartum bleeding, fetal growth restriction, fetal malformations, preterm delivery, and pre-eclampsia [141].

Zn deficiency may contribute to OS by elevating LPO levels due to diminished antioxidant defense mechanisms and compromised activity of Zn-dependent antioxidant enzymes, including Cu-Zn SOD [142,143]. Increased Cu/Zn ratios, resulting from imbalanced Cu and Zn levels, adversely impact the activity of antioxidant enzymes such as Cu/Zn SODs, ultimately leading to heightened LPO and impaired antioxidant defense systems, which have been implicated in the pathogenesis of pre-eclampsia. Consequently, Cu/Zn ratios may serve as potential predictive markers for vascular complications in pregnancies affected by pre-eclampsia [144].

Numerous studies have investigated the effects of Zn supplementation on clinical manifestations and metabolic status in patients with intrauterine growth restriction (IUGR). It has been observed that Zn levels in women with moderate and severe IUGR were significantly lower compared to women without IUGR [145]. Considering that Zn intake protects trophoblast cells from mitochondrial OS and inflammatory markers, it may hold importance in the treatment of women with IUGR [146]. Therefore, Zn supplementation may serve as an appropriate adjunct therapy for pregnant women at risk of IUGR [146].

Furthermore, administration of Zn at a dosage of 30 mg/day for 6 weeks to patients with GDM was found to have beneficial effects on metabolic profiles [147]. It should be noted that Zn was not identified as a causal mediator of the effects of other metals on OS [148].

### 5.4. Manganese

Manganese (Mn) is another essential element that plays a vital role in the synthesis and activation of various enzymes and in the regulation of glucose and lipid metabolism. It acts as an important cofactor for numerous enzymes, including the antioxidant Mn superoxide dismutase (Mn-SOD), which plays a role in protecting the placenta from OS by detoxifying O2• [149]. Some studies suggest that low Mn levels may reduce the activity of Mn superoxide dismutase, leading to the accumulation of reactive oxygen species and the development of pre-eclampsia [150,151].

### 5.5. Selenium

Se is an essential trace element that plays a critical role in the synthesis and function of endogenous antioxidants, such as GPx, selenoprotein P, thioredoxin reductase (TrxR), and iodothyronine deiodinases (IDD) [152]. It exerts control over the antioxidative activity of the enzymatic glutathione system [153], acts as an antioxidant, supporting both humoral and cell-mediated immunity, and is significant for reproductive processes [154].

Dietary Se is primarily bound to amino acids such as selenocysteine and selenomethionine. In organs such as the spleen, liver, serum, and blood, selenates (VI) undergo reduction to selenites (IV) or hydrogen selenide (H_2_Se). Selenates in the IV oxidation state exhibit higher tissue affinity, forming complexes with proteins and displaying enhanced incorporation into GPxs. The enzymatic activity of GPxs increases with elevated Se concentrations [155]. These compounds possess the capability to traverse the blood–placenta barrier, thereby reaching the fetal compartment.

Throughout pregnancy, there is a gradual decline in Se concentration due to increased placental transport and transfer to breast milk [156]. Studies have suggested that Se deficiency is associated with various pregnancy disorders, including miscarriage, pre-eclampsia, gestational diabetes mellitus, pregnancy-induced hypertension, neural tube defects, fetal growth restriction, and preterm birth [157,158,159,160,161]. Se deficiency, especially in the second trimester, has been found to be associated with OS and an increase in inflammatory mediators [162], affecting the risk of developing pregnancy disorders by reducing placental GPX activity and impeding the function of other Se-dependent antioxidants, including thioredoxin reductases (TXNRD), thereby leading to placental OS [163]. To optimize the antioxidative potential of GPX, serum Se levels should ideally reach approximately 100 μg Se/L [164]. Levels falling below this threshold in pregnant women may detrimentally affect fetal growth.

Supplementation with Se has demonstrated the ability to enhance cell proliferation, mitigate DNA damage, and attenuate apoptosis under normal conditions or in the presence of OS [165]. Administering Se during pregnancy holds promise for reducing maternal OS and yielding beneficial effects for both the mother and fetus [166]. Reported benefits of Se supplementation encompass a reduction in the incidence of pre-eclampsia/pregnancy-induced hypertension (PE/PIH), GDM, IUGR, preterm premature rupture of membranes (PROM), postpartum depression, and postpartum thyroid dysfunction. Furthermore, Se supplementation may influence breast milk composition, fetal lipid profile, and fetal bilirubin levels, although it has had mixed outcomes among HIV-positive mothers and their newborns [166].

Studies conducted on Iranian women from Arak reported favorable effects of Se supplementation on OS in pregnant women but did not observe reductions in the incidence of PE, FGR, and preterm birth [167,168,169]. That observation may be attributed to the small sample size of the studies conducted, and it should be noted that women from the Arak region may have higher Se concentrations compared to women from other regions of Iran.

Currently, the underlying mechanisms concerning the role of Se and Se-dependent enzymes remain unclear. Additionally, the optimal dosage, timing, and duration of Se supplementation during pregnancy are still subjects of ongoing debate and investigation [170].

### 5.6. Lead and Oxidative Stress

Lead is a common occupational and environmentally toxic metal in many industrialized countries, exhibiting nephro-, hepato-, osteo-, and neurotoxic effects [171]. ROS, including hydroperoxide, hydrogen peroxide, and singlet oxygen, are generated as a result of Pb poisoning [172]. Lead generates ROS, which leads to oxidative stress that causes cellular damage and can cause neurodegeneration and kidney and liver damage [173,174,175]. Oxidative stress as a mechanism of Pb toxicity in the kidney shows that Pb exposure causes an increase in apoptosis in the kidney [175]. Pb has been shown to inhibit the activity of 5-aminolevulinic acid dehydratase, resulting in hemoglobin oxidation and lipid peroxidation, which can lead to erythrocyte hemolysis [176]. Studies have shown that increased Pb levels lead to an imbalance between ROS and antioxidants in tissues and cellular components, causing damage to membranes, DNA, and proteins [174]. Pb has been shown to alter antioxidant activities by inhibiting functional sulfhydryl (SH) groups in several enzymes, such as SOD, CAT, GPx, and G6PD [177]. GPx, CAT, and SOD are potential targets for Pb toxicity because these antioxidant enzymes depend on several essential trace elements for proper molecular structure and activity [177]. Impaired antioxidant defenses may result from Pb’s inhibitory effects on various enzymes, which in turn makes cells more susceptible to oxidative insults. In addition, Pb can affect the bioavailability and absorption of trace elements, including Fe, Zn, and Cu. Zn and Pb compete for similar binding sites on the metallothionein-like transport protein, and this may reduce the absorption of Pb, thereby reducing Pb toxicity [177]. While Ca, Zn, and Fe cannot completely eliminate Pb from the body, they can reduce its levels.

### 5.7. Undernutrition and Oxidative Metabolism

Undernutrition leads to oxidative stress, which can disrupt oxidative homeostasis, activate a cascade of molecular pathways, and alter the metabolic status of various tissues [178]. Maternal nutritional status is a critical determinant of long-term health outcomes in offspring. Mothers suffering from malnutrition and antioxidant deficiency can create a situation of oxidative stress [179]. Pregnancy requires an increased intake of macro- and microelements (Zn, Cu, Se, and Fe supplementation) for maternal and fetal needs, and maternal undernutrition during pregnancy has been shown to lead to low birth weight and adverse perinatal outcomes [179,180,181]. WHO [182] noted that most low-birth-weight infants are born to undernourished mothers. In addition, Black et al. [183] found that maternal undernutrition contributes to fetal growth restriction, which increases the risk of neonatal death and, for survivors, stunting at 2 years of age. Aly et al. [184] found that nutritionally stunted children had increased oxidative stress and decreased antioxidant defenses compared to healthy controls. Oxidative stress may play an important role in the pathogenesis of stunting. 

In addition, undernutrition has long-lasting physiological effects, including increased susceptibility to fat accumulation, lower fat oxidation, lower resting and postprandial energy expenditure, adult insulin resistance, hypertension, and dyslipidemia [181]. Malnutrition affects the placenta, leading to deficient development or reduction in the enzymatic barrier 11-β-HSD, allowing excess cortisol to reach the fetus. The catabolic effects of cortisol are associated with increased ROS production [179]. Some studies have found that a low-protein diet during pregnancy increases oxidative stress biomarkers in the placenta and metabolic dysfunction (later chronic disease risk) in the offspring [179,185,186]. The relationship between undernutrition during pregnancy, maternal oxidative stress, and metabolic abnormalities in the offspring remains unclear and warrants further investigation.

## 6. Biomarkers of Oxidative Stress

Direct determination of ROS concentrations poses challenges due to their short half-lives. As a result, protein markers are employed to assess the extent of redox imbalance. These OS markers encompass molecules that undergo modifications as a consequence of ROS interactions, as well as components of the antioxidant system that undergo changes and modifications in response to stress conditions [187]. The selection of an OS marker relies on specific criteria, including its specificity, sensitivity to elevated ROS levels, temporal stability enabling sample collection and analysis, and result in reproducibility [188].

DNA, lipids (including phospholipids), proteins, and carbohydrates exemplify molecules susceptible to in vivo modifications induced by excessive ROS levels [188]. Hence, indirect assessment of ROS levels by examining oxidative damage to lipids, proteins, and nucleic acids within cellular systems presents a promising alternative for evaluating OS in clinical samples. An array of markers is employed to describe OS, encompassing MDA, nitric oxide (NO), ROS, TAC, total antioxidant activity (TAA), SOD, GPx, glutathione peroxidase-4 (GPx4), glutathione reductase (GR), LPO, 8-hydroxydeoxyguanosine (8-OHdG), oxidized glutathione (GSSG), CAT, superoxide (O_2_^-^), paraoxonase (PON-1), oxidative stress index (OSI), high-sensitivity C-reactive protein (hs-CRP), 8-iso-prostaglandin F2α (8-iso-PGF2α), prostaglandin F2α (PGF2α), GSH, and glutathione transferase (GST). These markers are analyzed using various materials, primarily blood (serum or plasma) and placenta, along with urine, Wharton’s jelly mesenchymal stem cells derived from the umbilical cord, and saliva [189,190].

Biomarkers of OS are employed in monitoring studies to assess pregnancy complications indirectly [191]. 

OS severity in cells and tissues is measured with, among others, LPO. Several factors contribute to increased LPO intensity, including elevated circulating lipoprotein levels, prooxidative activity of the placenta, and altered basal metabolism during pregnancy [192]. Among the OS used to evaluate LPO, MDA, a low-molecular-weight aldehyde resulting from the breakdown of polyunsaturated fatty acids, is widely utilized [193]. Laboratory quantification of MDA involves its reaction with thiobarbituric acid, providing a measure of OS. As a secondary product of LPO, MDA exerts cellular toxicity and can interact with DNA and proteins, often leading to mutagenesis. Additionally, MDA exhibits potential atherogenic properties. The reference concentration range of MDA in the blood serum of healthy individuals ranges from 0.32 to 53.8 nmol/mL; however, the genotoxicity associated with this LPO product should be considered [194]. Notably, physiological cellular metabolism can lead to endogenous MDA production, and its concentration is significantly influenced by factors such as diet, physical activity, and sample storage conditions [194]. Mentese et al. [195] demonstrated the high sensitivity of MDA as an indicator of neonatal hypoxia. Numerous studies have reported elevated MDA levels in serum, plasma, and placental tissue samples of preeclamptic women [40]. Rudra et al. [196] observed a correlation between a high plasma MDA concentration and the occurrence of pre-eclampsia. F2-isoprostanes, particularly 8-iso-PGF2α, are considered the most reliable in vivo indicators of LPO. 8-iso-PGF2α is enzymatically generated through the prostaglandin-endoperoxide synthase pathway. Extensive research involving nearly 500 animal studies and 900 human studies has demonstrated correlations between 8-iso-PGF2α and various diseases and exposures. Importantly, 8-iso-PGF2α differs from its enzymatic LPO analog [197]. The results of many studies indicate that TBARS, as an oxidative stress marker, may be used in clinical practice in the assessment of the severity of complications and as an indicator for timely delivery in women with pregnancy-induced hypertension (Table 1).

SOD serves as an example of an antioxidant system molecule that undergoes alterations and modifications in response to OS. Its concentration in the blood serum of healthy individuals is approximately 4315 U/mg. OS induces a sharp increase in SOD synthesis. Conversely, low or reduced SOD activity is associated with an elevated risk of OS-related pathological states [205]. Hernández-Trejo et al. [119] demonstrated higher levels of SOD, arginase, carbonylated proteins (CP), and nitrites in umbilical cord blood samples compared to maternal blood samples. But levels of GPx, MDA, LOOH, and free fatty acids (FFA) in newborns were lower than in their mothers. This may indicate a buffering role of OS via the placenta and suggests the involvement of various factors that influence the redox balance, as mentioned several times before. HIF1A serves as an indicator of hypoxia and plays a role in the cellular response to OS. Ashur-Fabian [206] found that maternal serum mRNA levels of HIF1A may reflect the hypoxic state during pregnancy, while HIF1A levels in the placenta better represent fetal hypoxia. Zhang et al. [207] observed significantly higher mRNA levels of HIF1A in the placenta of monochorionic twins with intrauterine growth restriction, particularly in fetuses with restricted growth. 

A total of 70–85% of pregnant women, especially early in the first trimester, may develop nausea and vomiting (NVP), which is closely related to oxidative stress. Verit et al. [208] found a strong relationship between oxidative stress and the clinical severity of NVP. The authors found that pregnant women with severe NVP had a higher total oxidant status (TOS) but a lower antioxidant status (TAS) than pregnant women with mild NVP. In addition, Rhodes’ index (which can assess the severity of the disease) showed a positive correlation with TOS and a negative relationship with TAS. Therefore, the authors suggest that TOS and TAS levels may serve as additional markers for the diagnosis and assessment of the clinical severity of NVP. In addition, Drejz et al. [189], based on an analysis of 83 selected papers, recommend the establishment of a common core panel of OS markers to be used in all studies related to OS in obstetrics and gynecology. They suggest including ROS as a direct marker of OS, 8-OHdG as a marker of DNA/RNA damage, and MDA as a marker of LPO. They also suggest including two commonly used antioxidant parameters, TAC and GSH, in the core panel of tests. However, they did not subject data from the scientific literature to statistical analysis.

## 7. Research on Animal Models

Due to the limitations of conducting direct studies on pregnant women, many research investigations are carried out using animal models. For example, Xu et al. [209] demonstrated the significant pro-oxidative effects of lipopolysaccharide (LPS) in the amniotic fluid during the embryonic development of rats. LPS exerted a substantial influence on the occurrence of external fetal abnormalities, intrauterine growth retardation, and fetal death. Substantiating the impact of OS, the administration of an antioxidant effectively mitigated these effects. 

Studies conducted in calves have indicated that cesarean sections lead to increased OS, characterized by elevated levels of malondialdehyde and reduced catalase activity. This phenomenon results in LPO and subsequent tissue damage [210].

Furthermore, the embryos of diabetic rats exhibit heightened free oxygen radical activity, which is believed to underlie the teratogenicity associated with diabetic pregnancies [211,212].

Guo et al. [213] investigated the effects of catalase supplementation on the activity of antioxidant enzymes and reproductive performance in sows and their offspring. Their findings demonstrated that administering catalase reduced the incidence of IUGR and improved antioxidant capacity in both sow serum and piglet umbilical cord samples. Viana et al. [214] provided evidence of oxidative DNA damage in rat embryos affected by diabetes, linking this mechanism to the teratogenic effects observed in the fetus. Chronic OS was found to contribute to a higher frequency of fetal resorption in rat models [215]. Cederberg and Eriksson [216] suggested that catalase may play a protective role against diabetic embryopathy in rats, while ROS are implicated as mediators of this teratogenic process. Similarly, Sivan et al. [217] demonstrated, in a rat model, that the excessive oxidative burden observed in diabetic pregnancies can result in embryopathy and tissue damage.

Supplementation with Se and Cr shows promise for preventing the development of GDM by alleviating endoplasmic reticulum stress in the liver.Studies utilizing laboratory animals have also indicated that zinc gluconate supplementation may improve symptoms and pregnancy outcomes in pre-eclampsia by mitigating OS and modulating the balance of systemic inflammatory responses and angiogenic factors [218].

## 8. Conclusions

The available scientific literature provides compelling evidence for a plausible association between compromised antioxidant enzyme activity and the occurrence of adverse pregnancy outcomes. OS exerts detrimental effects on maternal physiology, pregnancy progression, and fetal development by disrupting placental function and compromising oxygen and nutrient delivery to the developing fetus. This oxidative imbalance can contribute to miscarriages, fetal developmental abnormalities, preterm birth, and low birth weight. Consequently, it is crucial to further explore this field of research, particularly in conjunction with comprehensive investigations into the interplay of micronutrients and macronutrients. However, pregnant women should be cautious when taking trace element supplementation. This should advance our understanding of the underlying mechanisms and enable the development of effective interventions to mitigate the detrimental effects of OS during pregnancy.

The authors suggest that this panel should be created based on the results of other authors’ studies; however, the results were not subjected to statistical analysis. As there are no universal parameters to assess oxidative stress in human reproduction and pregnancy-related issues, they wanted to create a standardized set of parameters.

## Figures and Tables

**Table 1:** The thiobarbituric reactive substance (TBARS) values in non-pregnant, pregnant, and pre-eclampsia women.

Biological Materials	Group	Value	Reference
Plasma	healthy non-pregnant women (n = 50)	2.51 nmol/mL	Pasupathi et al. [198]
	health pregnant women (n = 50)	4.12 nmol/mL	
	pregnancy-induced hypertension (n = 50)	6.88 nmol/mL	
	normotensive controls pregnant women (n = 472)	6.7 µM	Mistry et al. [199]
	women with pre-eclampsia (n = 244)	6.5 µM	
	women with pre-eclampsia (n = 40)	3.8 µmol/L	Raijmakers et al. [200]
	normotensive pregnant controls matched for gestational age (n = 24)	1.5 µmol/L	
	pregnant women without hypertension (n = 100)	20 µmol (99% women)	Draganovic et al. [201]
	pregnant women with hypertension (n = 100)	20–40 µmol (66% women), 40 µmol (34% women)	
umbilical cord blood	normal pregnant (n = 27)	0.6 μmol/L	Mistry et al. [202]
	25 pre-eclamptic (n = 25)	0.8 μmol/L	
	healthy pregnant women (n = 42)	1.08 μmol/L	Catarino et al. [203]
	PE pregnant women (n = 46)	1.10 μmol/L	
amniotic fluid	pregnant women without hypertension (n = 100)	13.41 (96% women)–13.72 (4%) µmmol	Draganovic et al. [204]
	pregnant women with hypertension (n = 100)	35.17 (79%)–42.37 (21%) µmmol	
